# Glycosyltransferase GLT8D1 and GLT8D2 serve as potential prognostic biomarkers correlated with Tumor Immunity in Gastric Cancer

**DOI:** 10.1186/s12920-023-01559-y

**Published:** 2023-06-05

**Authors:** Huimei Xu, Ke Huang, Yimin Lin, Hang Gong, Xueni Ma, Dekui Zhang

**Affiliations:** 1 Department of Gastroenterology, The Second Clinical Medical College of Lanzhou University, Lanzhou, 730030 P.R. China; 2 Lanzhou University Second Hospital, Lanzhou, 730030 P.R. China; 3 School of Basic Medical Sciences, Lanzhou University, Lanzhou, 730030 P.R. China; 4 Key Laboratory of Digestive Diseases of Lanzhou University Second Hospital, Lanzhou, 730030 P.R. China

**Keywords:** Gastric cancer, GLT8D1, GLT8D2, Prognosis, Biomarker, Tumor immunity

## Abstract

**Background:**

Glycosylation involved in various biological function, aberrant glycosylation plays an important role in cancer development and progression. Glycosyltransferase 8 domain containing 1 (GLT8D1) and GLT8D2, as members of the glycosyltransferase family proteins, are associated with transferase activity. However, the association between GLT8D1/2 and gastric cancer (GC) remains unclear. We aimed to investigate the potential prognostic value and oncogenic role of GLT8D1/2 in GC.

**Methods:**

The relationship between GLT8D1/2 and GC was evaluated through comprehensive bioinformatics approaches. A series of factors like gene expression patterns, Kaplan-Meier survival analyses, Cox regression analyses, prognostic nomogram, calibration curves, ROC curves, function enrichment analyses, tumor immunity association, genetic alterations, and DNA methylation were included. Data and statistical analyses were performed using R software (v3.6.3).

**Results:**

Both GLT8D1 and GLT8D2 expression were significantly upregulated in GC tissues(n = 414) compared with normal tissues(n = 210), and high expression of GLT8D1/2 was remarkably correlated with poor prognosis for GC patients. Cox regression analyses implied that GLT8D1/2 could act as independent prognostic factors in GC. Furthermore, gene function analyses indicated that multiple signaling pathways involving tumor oncogenesis and development enriched, such as mTOR, cell cycle, MAPK, Notch, Hedgehog, FGF, and PI3K-Akt signaling pathways. Moreover, GLT8D1/2 was significantly associated with immune cell infiltration, immune checkpoint genes, and immune regulators TMB/MSI.

**Conclusion:**

GLT8D1/2 may serve as potential prognostic markers of poor prognosis in GC correlated with tumor immunity. The study provided an insight into identifying potential biomarkers and targets for prognosis, immunotherapy response, and therapy in GC.

**Supplementary Information:**

The online version contains supplementary material available at 10.1186/s12920-023-01559-y.

## Background

Gastric cancer (GC) remains one of the most common malignancies and the leading cause of cancer-related mortality worldwide. Despite there is a gradual decline in the incidence and mortality of stomach cancer over the past century, GC displays the highest incidence rates in Eastern Asia, Central and Eastern Europe, and South America [1, 2]. GC still elicits a serious health burden with more than one million new cases diagnosed each year globally [3, 4]. There are many treatment options for GC patients, including endoscopic resection, surgery, chemotherapy, radiotherapy. Especially, targeted therapy and immunotherapy, such as EGFR-targeted therapy, VEGF-targeted therapy, and anti-CTLA4/PD-1/PD-L1 immunotherapy have become available [1]. Regrettably, only a small percentage of cancer patients can benefit from immunotherapy or targeted therapy [5]. Advanced-stage GC patients following neoadjuvant chemoradiotherapy, targeted therapy, and immunotherapy are still correlated with a poor 5-year survival rate [6, 7], resistance and recurrence are still the main obstacles in the GC treatment [8]. Therefore, it is urgently important to identify more effective and reliable therapeutic targets and prognostic biomarkers for GC.

Glycosylation of proteins, a highly regulated and complex process, is crucial for cell adhesion, signaling, cell-cell communication, cell-matrix interaction, as well as response to the microenvironment. Aberrant glycosylation caused by alterations in glycosyltransferase activity is common in carcinoma cells and is usually related to cancer progression and metastasis. Glycosyltransferase 8 domain containing 1 (GLT8D1) and glycosyltransferase 8 domain containing 2 (GLT8D2), two novel glycosyltransferases, are vital for cell adhesion and cell-cell communication. Several studies have implied that GLT8D1 dysfunction is linked to neurodegenerative or neurological diseases, such as amyotrophic lateral sclerosis (ALS), frontotemporal dementia (FTD), and schizophrenia [9–13]. However, few reports have been published regarding the association between GLT8D1 and cancer. GLT8D1 is reported to play a tumorigenic role in head and neck squamous cell carcinomas and human cutaneous melanomas [14, 15]. Two recent studies revealed that GLT8D1 is associated with cell cycle arrest, apoptosis, growth, and self-renewal of glioma stem cells, and promotes migration of human glioblastoma cells. High-expressed GLT8D1 is confirmed to be correlated with worse clinical outcomes in glioma and glioblastoma [16, 17]. And limited research focused on GLT8D2. A pivotal role of GLT8D2 in non-alcoholic fatty liver disease pathogenesis was investigated [18, 19]. Additionally, overexpression of GLT8D2 confers ovarian cancer to cisplatin (CDDP) resistance. GLT8D2 is a potential therapeutic target, which may enhance the sensitivity to platinum for ovarian cancer patients with chemoresistance [20]. Association between GLT8D1/2 and the development of GC was not described so far, the biological functions and molecular mechanisms of GLT8D1/2 in GC remain unknown. Therefore, we conducted the bioinformatic analysis by combining multiple databases to comprehensively explore the roles of GLT8D1/2 in GC.

In the present study, for the first time, we visualized the expression and prognostic landscape, gene functions, tumor immunity correlation, genetic alterations, and DNA methylation of GLT8D1/2 in GC. Although further experimental validation is required to decipher potential values and molecular mechanisms of GLT8D1/2, this study proposed an insight into the pathogenesis or clinical prognosis of GC.

## Methods

### Data collection and integration

RNA-seq data and the corresponding clinicopathologic information were obtained from TCGA and GTEx databases. Data was standardized through the Toil procedure into TPM (transcripts per million reads) format from UCSC XENA (https://xenabrowser.net/datapages/), then performed log2 transformation [21]. TCGA data included 414 GC samples and 36 cancer-adjacent samples, and GTEx included 174 normal samples. Clinical characteristics of GC samples were analyzed between high expression and low expression of GLT8D1/2 based on median expression levels. Samples without available or clear clinical information were excluded.

### Gene expression analysis

GLT8D1/2 mRNA expression signature was analyzed across the 33 TCGA cancers using Mann-Whitney U (Wilcoxon rank sum test) and visualized by ggplot2 package (version 3.3.3) of R software (version 3.6.3). Especially, the difference of GLT8D1/2 mRNA expression in GC tissues compared with normal tissues (including 174 normal tissue samples in GTEx and 36 cancer-adjacent samples in TCGA) was analyzed and presented. In addition, the correlation between GLT8D1/2 expression and different clinicopathologic characteristics in GC was also analyzed. *P* < 0.05 was considered statistically significant.

### Kaplan-meier survival analysis

The association between GLT8D1/2 and overall survival (OS), disease-specific survival (DSS), and recurrence-free survival (RFS) prognosis of GC patients was performed using Kaplan-Meier Plotter (http://www.kmplot.com/analysis/index.php?p=service&cancer=96pancancer_rnaseq) and R software (version 3.6.3) with packages of “survminer (version 0.4.9) and survival (version 3.2.10)” based on TCGA. The prognostic data were obtained from the study published in Cell [22]. All GC patients were grouped into high-expression and low-expression GLT8D1/2 according to the median expression with a 50% cutoff. Hazard ratio (HR) with 95% confidence interval (95% CI) and logrank test were calculated in the hypothesis test, logrank *P*-value < 0.05 was considered a significant difference.

### Cox regression analyses

Univariate and multivariate Cox regression analyses were conducted to evaluate whether GLT8D1/2 expression, gender, age, pathologic stage, Helicobacter pylori (H. pylori) infection, histologic grade, and residual tumor with OS were independent prognostic factors in GC patients. Cox regression analyses were performed by R software (version 3.6.3) with the “survival” package (version 3.2.10) based on the TCGA database. HR and 95% CI were calculated, *P*-value < 0.05 was statistically significant.

### Construction of prognostic nomogram, calibration plot, and ROC curves

The prognostic nomogram and calibration plot were constructed using R with “rms” package (version 6.2.0) and “survival” package (version 3.2.10) based on multivariate Cox regression analysis. Nomogram was applied as a prediction model to predict individualized gastric cancer prognosis by combining clinical characteristics and risk scores of the patients. The calibration plot was used to evaluate and verify the prediction accuracy of the prognostic nomogram. In addition, the diagnostic value of GLT8D1/2 expression was evaluated using Receiver Operating Characteristic (ROC) curves. The ROC curves were generated using R with the “pROC” package (version 1.17.0.1) and the “ggplot2” package (version 3.3.3). These curves provide a graphical representation of the sensitivity and specificity of GLT8D1/2 expression as a diagnostic marker. On the ROC curve plots, the horizontal axis represents the false positive rate (FPR), while the vertical axis represents the true positive rate (TPR).

### Function enrichment analysis of GLT8D1/2

Protein-protein interaction (PPI) was performed from the STRING database (https://cn.string-db.org/) to obtain GLT8D1/2-binding proteins. In addition, genes correlated with GLT8D1/2 expression were obtained using R with “stat” package (version 3.6.3). The correlation coefficient and Pearson *P*-value were calculated. The correlation between GLT8D1/2 and the selected top co-expressed genes was visualized by heatmaps and chord plots [23] using R packages (ggplot2, version 3.3.3 and circlize, version 0.4.121).

Moreover, Gene Ontology (GO) and Kyoto Encyclopedia of Genes and Genomes (KEGG) enrichment analyses [24–26] were performed on the GLT8D1/2-related genes obtained from the STRING database and single-gene correlation analysis. These analyses were conducted using R packages (“clusterProfiler 3.14.3” and “org.Hs.eg.db 3.10.0”) [27]. GO enrichment analysis included three domains: biological process (BP), cellular component (CC), and molecular function (MF). GO/KEGG analyses were visualized by bubble plots and network plots using R with “ggplot2” package. *P*-value < 0.05 was considered statistically significant.

### GSEA analyses of GLT8D1/2-related signaling pathways

Differentially expressed genes (DEGs) between high-expressed and low-expressed groups of GLT8D1/2 were analyzed using “DESeq2” package (version 1.26.0) in R [28]. After obtaining these DEGs, gene set enrichment analysis (GSEA) was utilized to explore potential signaling pathways [29]. Curated gene sets (c2.cp.v7.2.symbols.gmt) in MSigDB Collections (https://www.gsea-msigdb.org/gsea/msigdb/collections.jsp#C2) were selected as the reference for gene sets. GSEA was performed by R packages (clusterProfiler, version 3.14.3 and ggplot2, version 3.3.3). In GSEA, the gene sets with absolute value of Normalized Enrichment Score (NES) > 1, false discovery rate (FDR) < 0.25, and *p*.adjust < 0.05 were considered significantly enriched.

### Tumor immunity analyses

The correlation between GLT8D1/2 expression and immune cells infiltration, cancer-associated fibroblasts (CAFs) in GC were analyzed using TIMER 2.0 database [30] and R. Firstly, the correlation between GLT8D1/2 expression and 6 immune cells including B cell, CD8 + T cell, CD4 + T cell, macrophage, neutrophil, and dendritic cell was analyzed using “immune-gene” module of TIMER database (https://cistrome.shinyapps.io/timer/). Meanwhile, CAFs infiltration level was estimated through EPIC, MCPCOUNTER, and TIDE algorithms based on TIMER 2.0 database. Partial correlation (cor) and *P*-value were calculated via Spearman correlation test. The Scatter plots were visualized. Furthermore, the relationship between GLT8D1/2 expression and 24 types of immune cells [31] in GC was investigated using ssGSEA algorithm of R package (GSVA, version 1.34.0) [32]. Spearman correlation was analyzed, and *P* < 0.05 was considered significantly different.

In addition, the correlations between GLT8D1/2 expression and immune checkpoint genes, as well as immunomodulators such as tumor mutational burden (TMB) and microsatellite instability (MSI) were analyzed in GC. These immune checkpoint genes include CD274 (PD-L1), CTLA4, HAVCR2, LAG3, PDCD1 (PD-1), PDCD1LG2 (PD-L2), SIGLEC15, and TIGIT, playing an important role in tumor immune evasion [33–35]. The expression values of immune checkpoint-related genes were extracted based on TCGA database and correlation analyses were implemented by R software. The heatmap was presented. *P* < 0.05 indicated statistically significant.

To conduct the correlation analyses between GLT8D1/2 and TMB/MSI, RNA-sequencing expression profiles and corresponding clinical information for GC were downloaded from the TCGA dataset. Spearman’s correlation analysis was performed using R package ggstatsplot (https://github.com/IndrajeetPatil/ggstatsplot). High TMB with more neoantigens may indicate an improved response to treatment with immune checkpoint blockade [36]. High MSI levels exhibit a better anti-tumor response, the ability to inhibit tumor cell growth, and a better prognosis [37, 38]. *P* value < 0.05 was considered statistically significant.

### Genetic alteration analysis and DNA methylation of GLT8D1/2

We investigated the genetic alteration characteristics of GLT8D1/2 in pan-cancer by using cBioPortal web (https://www.cbioportal.org/) [39]. The alteration frequency and mutation type across all TCGA tumors were observed. The correlation between gene alterations in GLT8D1/2 and OS prognosis of GC patients was analyzed to identify its prognostic value. The survival data of GC cases with or without GLT8D1/2 genetic alteration was obtained by using the “comparison/survival” module of cBioPortal. Kaplan-Meier plots with log-rank *P*-value were presented.

Furthermore, GLT8D1/2 methylation levels in GC and cancer survival relationship were analyzed using MethSurv online tool (https://biit.cs.ut.ee/methsurv/) [40]. We analyzed GLT8D1/2 methylation in GC using “gene visualization” module to generate heatmap. DNA methylation values were represented as Beta values ranging from 0 (unmethylated) to 1(fully methylated). Then we performed survival analyses of individual CpG methylation based on “single CpG” module to evaluate the prognostic value of the GLT8D1/2 methylation in GC.

## Results

### Clinical characteristics of GC

375 GC tissues with clear baseline characteristics were obtained from TCGA database (https://portal.gdc.cancer.gov/). Clinical information included age, gender, TNM stage, H. pylori infection, histologic grade, residual tumor, and survival prognosis. The results showed that GLT8D1 expression was significantly correlated with age, pathologic stage, OS and DSS prognosis, while GLT8D2 expression was correlated with T stage, histologic grade, and OS prognosis (Table 1 and Table 2).

**Table 1:** Clinical characteristics of GC patients according to GLT8D1 expression

Characteristic	Low expression of GLT8D1	High expression of GLT8D1	*P*
n	187	188	
Gender, n (%)			0.884
Female	68 (18.1%)	66 (17.6%)	
Male	119 (31.7%)	122 (32.5%)	
T stage, n (%)			0.111
T1	14 (3.8%)	5 (1.4%)	
T2	40 (10.9%)	40 (10.9%)	
T3	88 (24%)	80 (21.8%)	
T4	44 (12%)	56 (15.3%)	
N stage, n (%)			0.189
N0	63 (17.6%)	48 (13.4%)	
N1	48 (13.4%)	49 (13.7%)	
N2	39 (10.9%)	36 (10.1%)	
N3	30 (8.4%)	44 (12.3%)	
M stage, n (%)			0.253
M0	165 (46.5%)	165 (46.5%)	
M1	9 (2.5%)	16 (4.5%)	
Pathologic stage, n (%)			0.032
Stage I	32 (9.1%)	21 (6%)	
Stage II	64 (18.2%)	47 (13.4%)	
Stage III	68 (19.3%)	82 (23.3%)	
Stage IV	14 (4%)	24 (6.8%)	
H. pylori infection, n (%)			0.781
No	71 (43.6%)	74 (45.4%)	
Yes	10 (6.1%)	8 (4.9%)	
Histologic grade, n (%)			0.808
G1	4 (1.1%)	6 (1.6%)	
G2	70 (19.1%)	67 (18.3%)	
G3	109 (29.8%)	110 (30.1%)	
Residual tumor, n (%)			0.173
R0	158 (48%)	140 (42.6%)	
R1	5 (1.5%)	10 (3%)	
R2	6 (1.8%)	10 (3%)	
OS event, n (%)			< 0.001
Alive	132 (35.2%)	96 (25.6%)	
Dead	55 (14.7%)	92 (24.5%)	
DSS event, n (%)			0.003
Alive	147 (41.5%)	116 (32.8%)	
Dead	34 (9.6%)	57 (16.1%)	
Age, n (%)			0.032
<=65	93 (25.1%)	71 (19.1%)	
> 65	93 (25.1%)	114 (30.7%)	

**Table 2:** Clinical characteristics of GC patients according to GLT8D2 expression

Characteristic	Low expression of GLT8D2	High expression of GLT8D2	*P*
n	187	188	
Gender, n (%)			0.617
Female	64 (17.1%)	70 (18.7%)	
Male	123 (32.8%)	118 (31.5%)	
T stage, n (%)			< 0.001
T1	18 (4.9%)	1 (0.3%)	
T2	43 (11.7%)	37 (10.1%)	
T3	84 (22.9%)	84 (22.9%)	
T4	42 (11.4%)	58 (15.8%)	
N stage, n (%)			0.473
N0	53 (14.8%)	58 (16.2%)	
N1	51 (14.3%)	46 (12.9%)	
N2	43 (12%)	32 (9%)	
N3	34 (9.5%)	40 (11.2%)	
M stage, n (%)			1.000
M0	167 (47%)	163 (45.9%)	
M1	13 (3.7%)	12 (3.4%)	
Pathologic stage, n (%)			0.073
Stage I	35 (9.9%)	18 (5.1%)	
Stage II	49 (13.9%)	62 (17.6%)	
Stage III	75 (21.3%)	75 (21.3%)	
Stage IV	20 (5.7%)	18 (5.1%)	
H. pylori infection, n (%)			0.302
No	95 (58.3%)	50 (30.7%)	
Yes	9 (5.5%)	9 (5.5%)	
Histologic grade, n (%)			0.011
G1	5 (1.4%)	5 (1.4%)	
G2	82 (22.4%)	55 (15%)	
G3	96 (26.2%)	123 (33.6%)	
Residual tumor, n (%)			0.623
R0	157 (47.7%)	141 (42.9%)	
R1	6 (1.8%)	9 (2.7%)	
R2	8 (2.4%)	8 (2.4%)	
OS event, n (%)			0.038
Alive	124 (33.1%)	104 (27.7%)	
Dead	63 (16.8%)	84 (22.4%)	
DSS event, n (%)			0.393
Alive	137 (38.7%)	126 (35.6%)	
Dead	42 (11.9%)	49 (13.8%)	
Age, n (%)			0.553
<=65	78 (21%)	86 (23.2%)	
> 65	106 (28.6%)	101 (27.2%)	

### Gene expression patterns at mRNA level

**Fig. 1:**
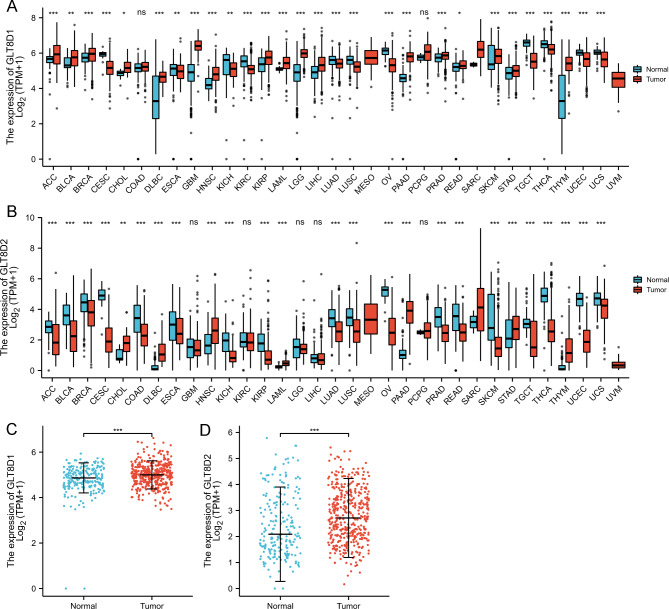
The mRNA expression levels of GLT8D1/2 in human pan-cancer and gastric cancer from the TCGA project. **(A, B)** The mRNA expression of GLT8D1 **(A)** and GLT8D2 **(B)** in pan-cancer compared with the corresponding normal tissues. **(C, D)** The mRNA expression level of GLT8D1 **(C)** and GLT8D2 **(D)** in gastric cancer compared with the normal tissues. ns, no significance; *P < 0.05; ** *P* < 0.01; *** *P* < 0.001

Our results demonstrated that GLT8D1 was significantly upregulated in the tumor tissues than the corresponding normal tissues in more than half of cancer types (17 out of 33) (Fig. 1A). Conversely, GLT8D2 expression presented a significantly lower level in more than half of cancer types (18 out of 33) (Fig. 1B). Interestingly, both GLT8D1 and GLT8D2 expression were upregulated consistently in GC compared with the normal tissues (Fig. 1 C and 1D).

Furthermore, the relationship of GLT8D1/2 expression with clinicopathological features in GC was analyzed. Different GLT8D1 expression was observed in groups based on residual tumor, with higher GLT8D1 in R1&R2 than that in R0. GLT8D2 expression was significantly different in pathologic stage, histologic grade, and T stage. For T stage, GLT8D2 expression of patients with T3&T4 was higher than T1&T2. Patients with high histologic grade (G3) showed higher GLT8D2 expression than grade G2. Additionally, GLT8D2 expression was higher in pathologic stage II/III than stage I. No differences were observed between GLT8D1/2 and other clinicopathological characteristics including H. pylori infection, M stage, and N stage (Figure S1).

### Survival analysis of GLT8D1/2 in GC

**Fig. 2:**
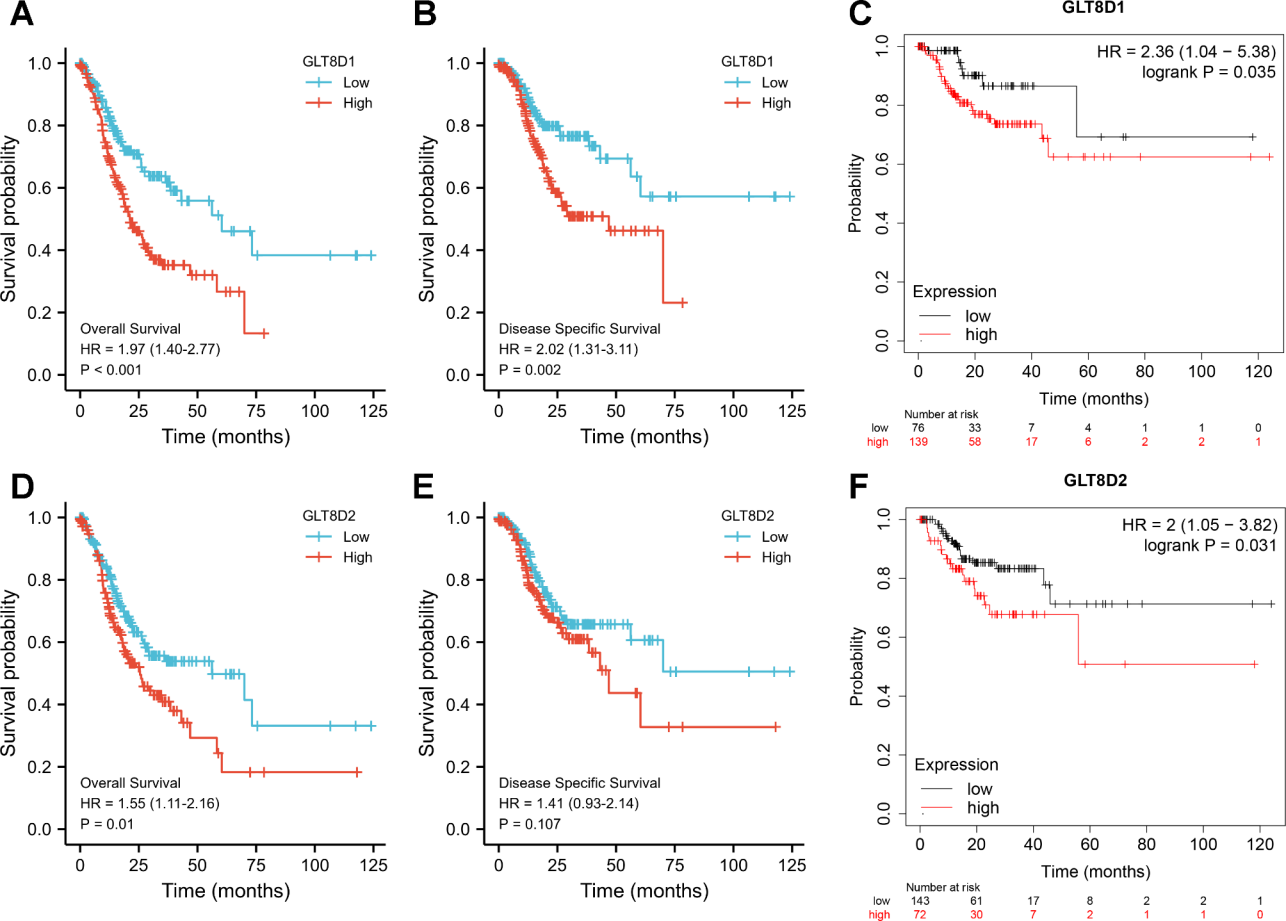
Kaplan-Meier survival curves of GLT8D1/2 in gastric cancer. **(A-C)** Survival curves of OS **(A)**, DSS **(B)**, and RFS **(C)** for patients divided into high-expression and low-expression of GLT8D1. **(D-F)** Survival curves of OS **(D)**, DSS **(E)**, and RFS **(F)** for patients divided into high-expression and low-expression of GLT8D2 in gastric cancer. OS, overall survival; DSS, disease-specific survival; RFS, recurrence-free survival; HR, hazard ratio

All cancer cases were divided into two groups based on the median gene expression level of GLT8D1/2. These groups were classified as high-expression and low-expression GLT8D1/2 groups. As shown in Fig. 2A, B, and C, the Kaplan-Meier curves consistently implied that GLT8D1 played a detrimental role in GC. High expression of GLT8D1 was significantly related to an unfavorable prognosis for OS (HR = 1.97, 95%CI, 1.40–2.77, logrank *P* < 0.001), DSS (HR = 2.02, 95%CI, 1.31–3.11, logrank *P* = 0.002), and RFS (HR = 2.36, 95%CI, 1.04–5.38, logrank *P* = 0.035). Patients with higher GLT8D2 expression showed a significantly worse OS (HR = 1.55, 95%CI, 1.11–2.16, logrank *P* = 0.01) (Fig. 2D) and RFS (HR = 2, 95%CI, 1.05–3.82, logrank *P* = 0.031) (Fig. 2F). Higher GLT8D2 was linked to poor DSS, but the difference was not statistically significant (HR = 1.41, 95%CI, 0.93–2.14, logrank *P* = 0.107) (Fig. 2E). Overall, high expression level of GLT8D1/2 was related to a poor prognosis for GC.

### Prognostic and diagnostic value of GLT8D1/2

**Fig. 3:**
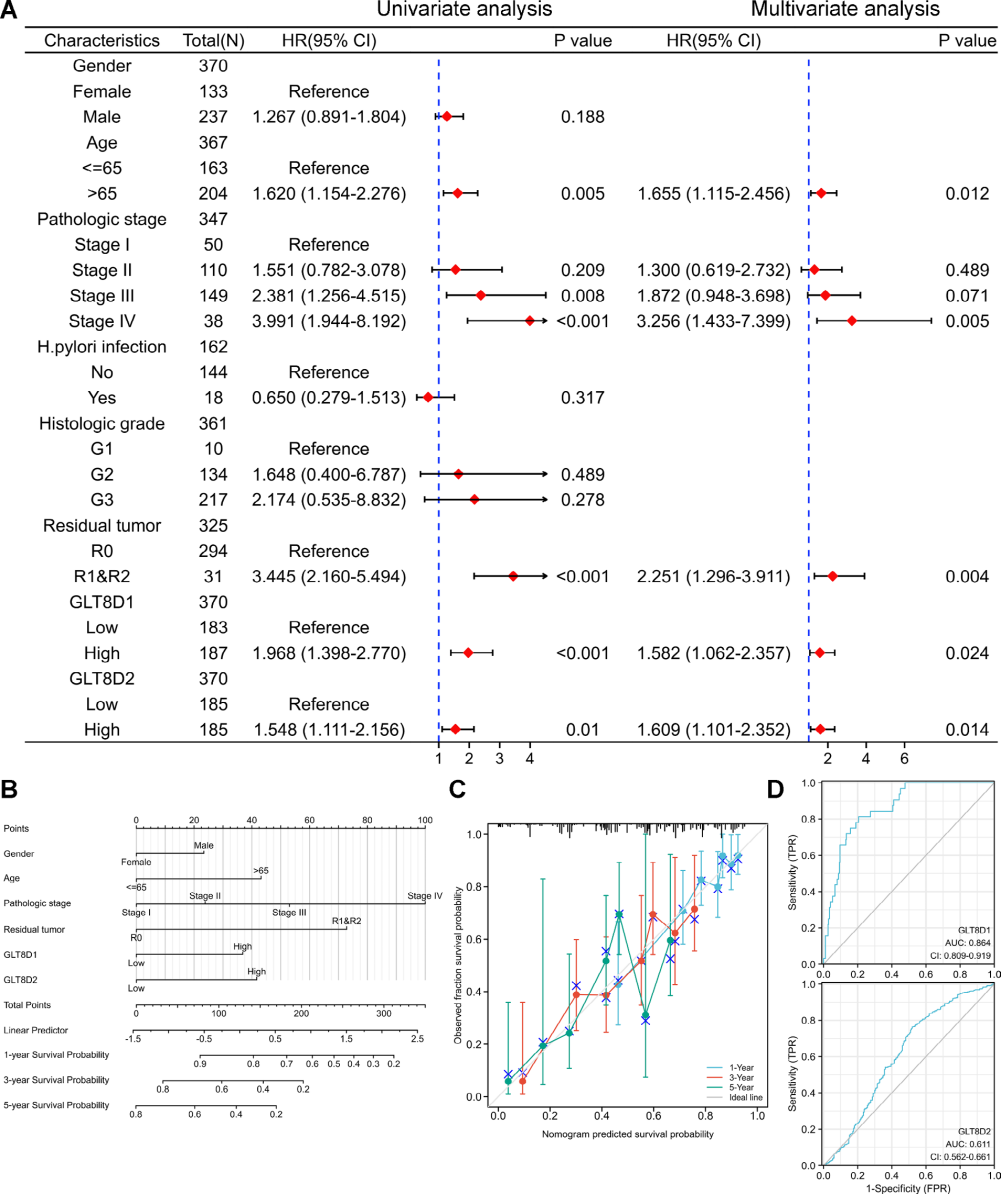
Prognostic and diagnostic value of GLT8D1/2 in gastric cancer. **(A)** Univariate and multivariate Cox regression analyses of GLT8D1/2 mRNA expression associated with OS prognosis in gastric cancer with different clinicopathological characteristics. Red squares indicate HR, HR > 1 with 95% CI greater than 1 indicates disadvantageous factors, and HR < 1 with 95% CI lower than 1 indicates protective factors. **(B)** The prognostic nomograms. **(C)** The calibration plot evaluated and verified the prediction accuracy of the prognostic nomogram. **(D)** The diagnostic value of GLT8D1/2 expression was evaluated using the ROC curves. The AUC value ranges between 0.5 (random-quality prediction) and 1 (perfect prediction), and the AUC value closer to 1, indicates a more accurate diagnostic model. ROC, receiver operating characteristic; AUC, area under ROC curve; HR, hazard ratio; FPR, false positive rate; TPR, true positive rate

Univariate regression analysis revealed that several clinical characteristics were related to the OS of GC patients, including age, pathologic stage III&IV, residual tumor, and higher GLT8D1/2 expression (Fig. 3A). Notably, after screening by multivariate regression analysis, the results showed that clinical parameters including age, pathologic stage IV, residual tumor, and higher GLT8D1/2 expression, were identified as independent prognostic factors for GC patients in this study (Fig. 3A). Next, we constructed the prognostic nomograms with prognosis factors including gender, age, pathologic stage, residual tumor, GLT8D1/2 expression to predict the 1-, 3- and 5-year survival probability (Fig. 3B). The calibration plot demonstrated that the predicted survival probability generated by the nomogram deviates to some extent from the ideal reference line, particularly in the case of 5-year survival. This observation suggests that the nomogram might exhibit reduced accuracy or reliability when making predictions for longer-term outcomes (Fig. 3C). The above results suggested that high expression of GLT8D1/2 may serve as independent risk factors for the poor prognosis of GC.

Furthermore, diagnostic value of GLT8D1/2 mRNA expression was evaluated by ROC curves. The AUC value of GLT8D1 was 0.864 indicating a good accuracy for diagnosis, while AUC of GLT8D2 presented low-quality diagnostic performance (AUC = 0.611) (Fig. 3D).

### Gene functions enrichment analysis of GLT8D1/2

**Fig. 4:**
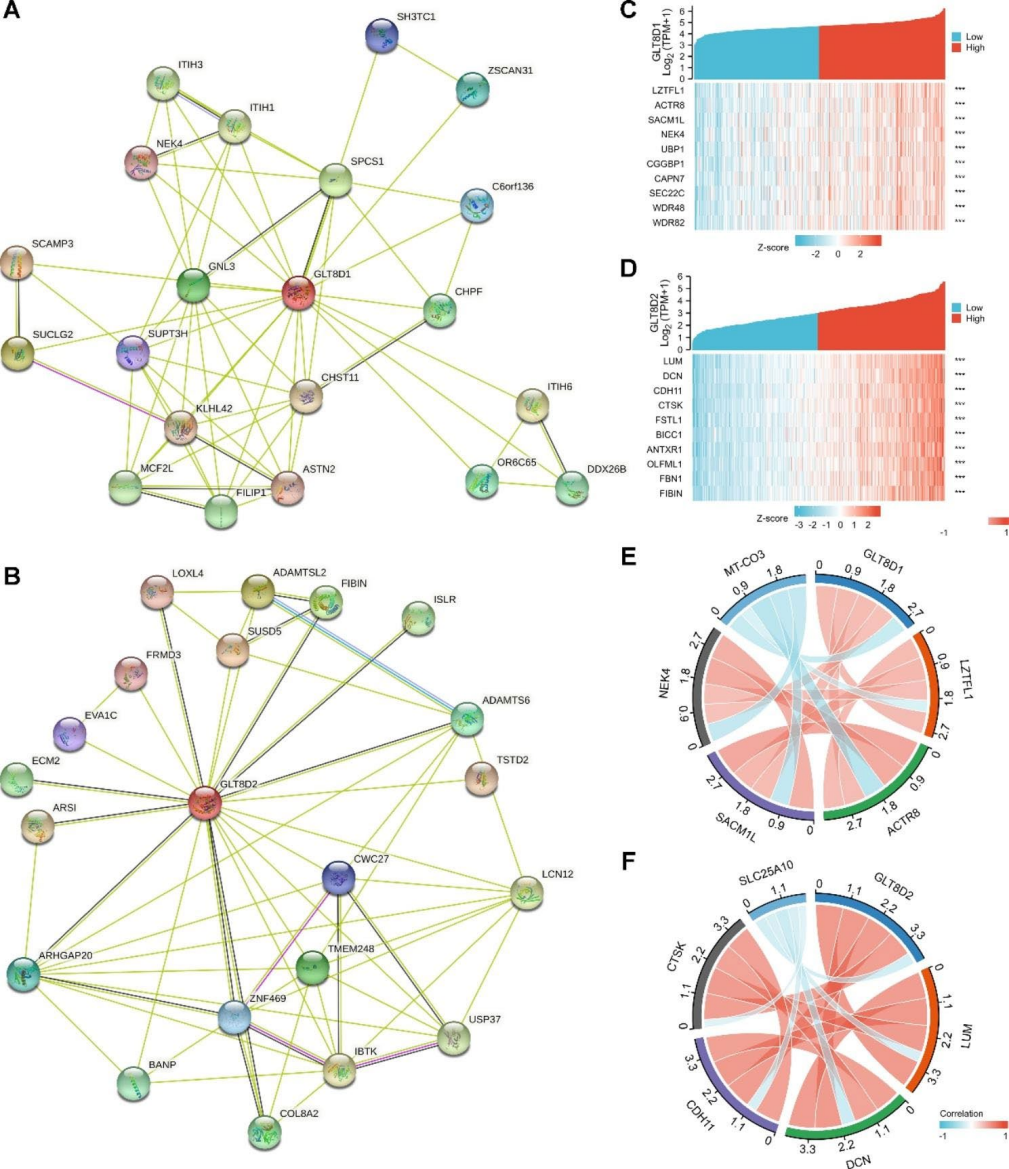
Protein-protein interaction (PPI) networks and molecular correlation analyses of GLT8D1/2. **(A, B)** PPI networks of 20 interacting proteins correlated with GLT8D1 **(A)** and GLT8D2 **(B)** based on the STRING tool. **(C)** The top 10 genes related to GLT8D1 expression were visualized in co-expressed gene heatmaps. **(D)** The top 10 genes related to GLT8D2 expression were visualized in co-expressed gene heatmaps. **(E)** Chord plots of the top 4 positively correlated genes and 1 negatively correlated gene of GLT8D1. **(F)** Chord plots of the top 4 positively correlated genes and the top 1 negatively correlated gene of GLT8D2. *** *P* < 0.001

In the study, we obtained the top 20 interacting proteins from the STRING tool, and PPI networks were presented in Fig. 4A and Fig. 4B. In addition, gene correlation analysis of GLT8D1/2 by R showed that 4948 genes were positively correlated with GLT8D1, and 26 genes were negatively correlated with GLT8D1. In parallel, 4348 genes showed a positive correlation with GLT8D2, while 122 genes showed a negative correlation with GLT8D2. The correlation between GLT8D1/2 and the selected top 10 positively related genes were visualized in co-expressed gene heatmaps (Fig. 4C and Fig. 4D). Chord plots presented the intercorrelations between GLT8D1/2 and the top 4 positively correlated genes and 1 negatively correlated gene (Fig. 4E, F).

**Fig. 5:**
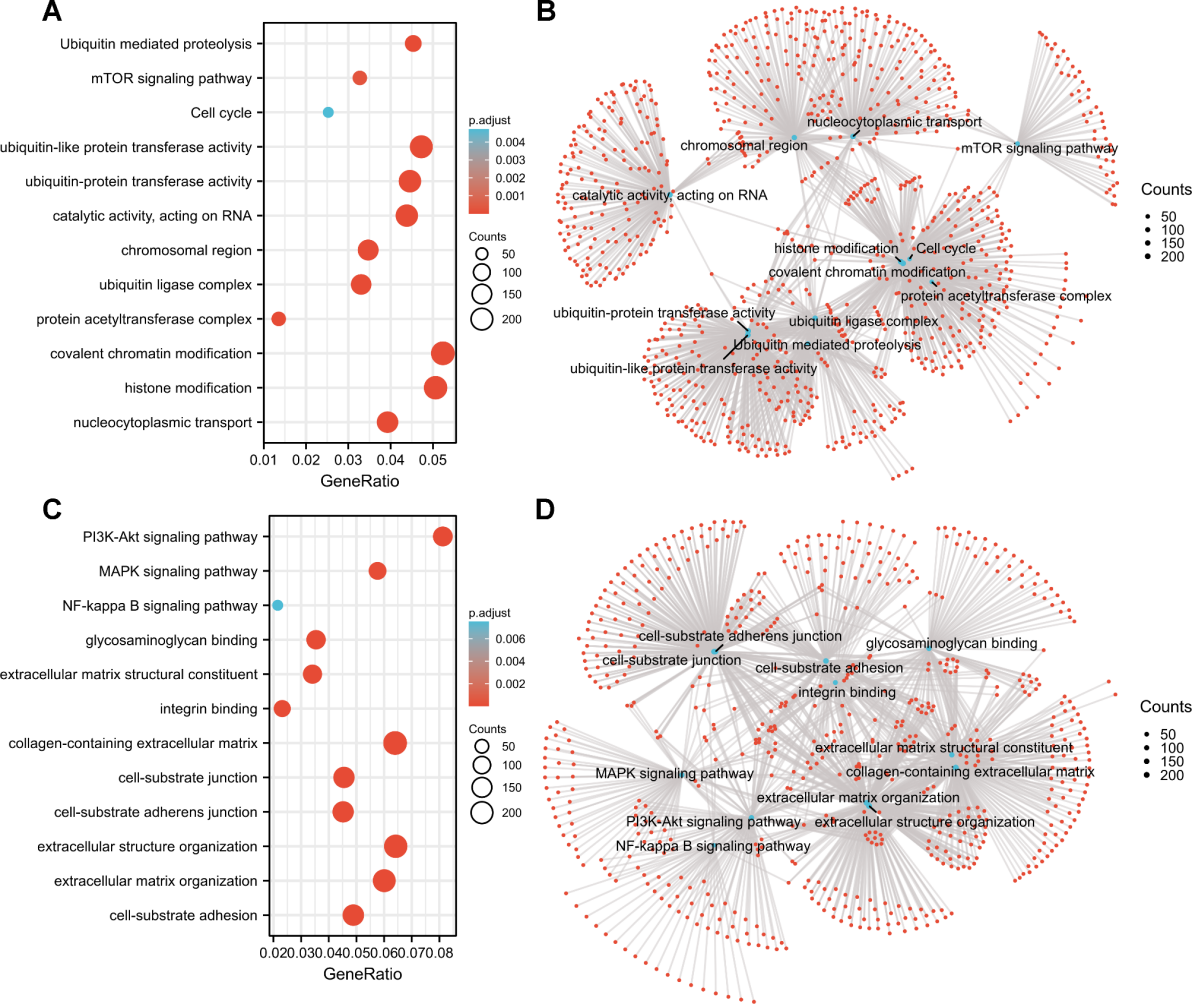
GO/KEGG functional enrichment analyses. **(A, B)** GLT8D1-related genes was presented as a bubble plot **(A)** and network visualization **(B)**. **(C, D)** GLT8D2-related genes was presented as a bubble plot **(C)** and network visualization **(D)**

Moreover, GO/KEGG enrichment analyses suggested that GLT8D1-related genes may be involved in covalent chromatin and histone modification, nucleocytoplasmic transport, protein acetyltransferase complex, protein ubiquitin, RNA metabolism, mTOR signaling pathway, and cell cycle, etc. (Figs. 5 A, 5B, and Table S1). GLT8D2-related genes were linked to extracellular matrix organization, cell-substrate adhesion and junction, glycosaminoglycan binding, integrin binding, PI3K-Akt, MAPK, NF-kappa B signaling pathways, and others (Fig. 5 C, 5D, and Table S2). The aforementioned results suggested that GLT8D1/2 expression was associated with multiple pathways or cellular biology involving tumor pathogenesis and development.

**Fig. 6:**
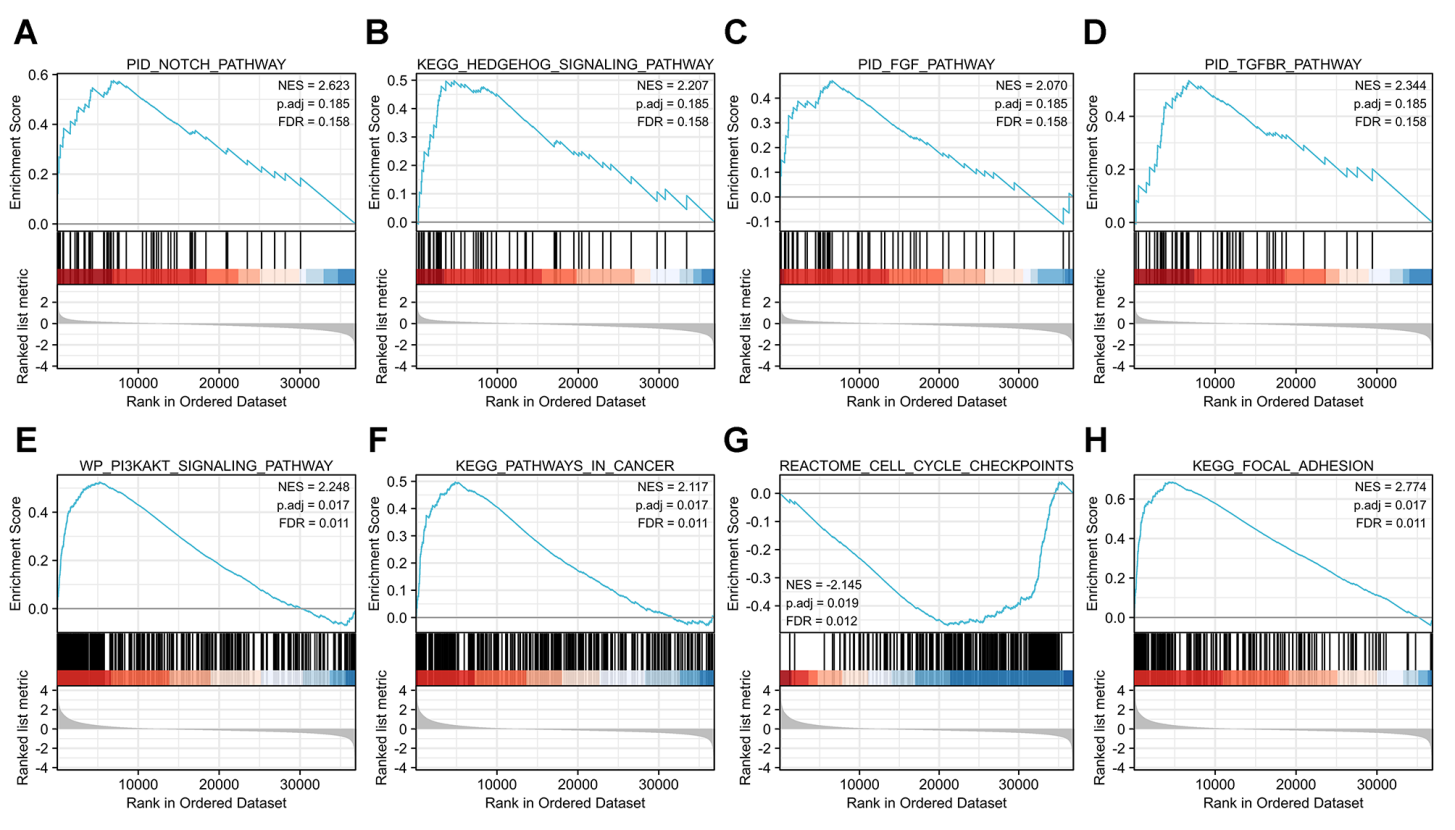
Gene set enrichment analysis (GSEA). **(A-D)** Four signaling pathways correlated with GLT8D1 enriched. **(E-H)** Four signaling pathways correlated with GLT8D2 enriched

Accordingly, GSEA analysis showed that GLT8D1/2 expression was significantly associated with multiple signaling pathways involving oncogenesis and tumor development. For example, Notch, Hedgehog, FGF, and TGFBR pathways correlated with GLT8D1 (Fig. 6A-D), while GLT8D2 was significantly linked to PI3K-Akt signaling pathways, pathways in cancer, cell cycle checkpoints, and focal adhesion (Fig. 6E-H). The gene functions enrichment analyses indicated that GLT8D1/2 potentially play vital roles in GC and provided new ideas for in-depth investigation.

### Tumor immunity correlation

#### Immune cells infiltration

**Fig. 7:**
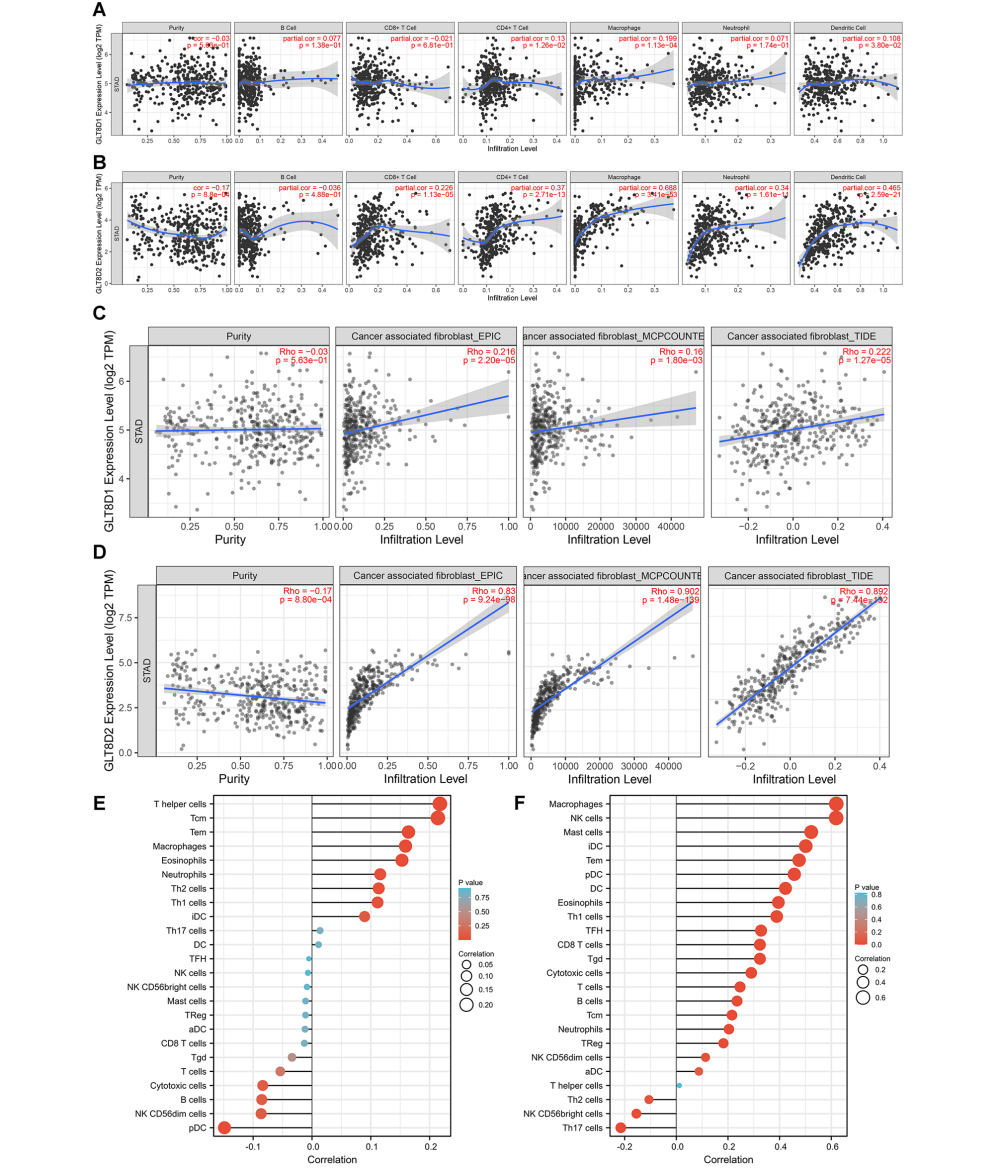
Correlation between GLT8D1/2 expression and infiltration of immune cells and CAFs in gastric cancer. **(A, B)** The correlation of GLT8D1 **(A)** and GLT8D2 **(B)** with six types of immune cells, including B cell, CD8 + T cell, CD4 + T cell, macrophage, neutrophil, and dendritic cell in the TIMER database. **(C, D)** The relationship of GLT8D1 **(C)** and GT8D2 **(D)** with CAFs infiltration using three algorithms of EPIC, MCPCOUNTER, and TIDE. **(E, F)** The correlation of GLT8D1 **(E)** and GLT8D2 **(F)** with 24 immune cell subtypes. CAFs, cancer-associated fibroblasts

To begin, we found that GLT8D1 expression was positively correlated with CD4 + T cell, macrophage, and dendritic cell in GC. No significant correlations of GLT8D1 with B cell, CD8 + T cell, and neutrophil were observed (Fig. 7A). GLT8D2 expression showed positive correlations with immune cell infiltration of CD8 + T cell, CD4 + T cell, macrophage, neutrophil, and dendritic cell, but no statistical correlation with B cell (Fig. 7B). Then we observed a significant positive correlation between GLT8D1/2 expression and CAFs infiltration level in GC based on EPIC, MCPCOUNTER, and TIDE algorithms (Fig. 7 C and 7D).

In addition, we explored 24 types of immune cells based on the TCGA. Results indicated that GLT8D1 expression had a significantly positive correlation with immune infiltration of T helper cells, Central Memory T cell (Tcm), Effector Memory T cell (Tem), Macrophages, Eosinophils, Neutrophils, T helper Type 1 cells (Th1 cells), and T helper Type 2 cells (Th2 cells), but a negative correlation with plasmacytoid dendritic cells (pDC) (Fig. 7E and Table S3). GLT8D2 expression was positively correlated with most immune cells (19 out of 24), including Macrophages, Natural Killer cells (NK cells), Mast cells, immature dendritic cells (iDC), Tem, pDC, dendritic cells (DC), Eosinophils, Th1 cells, T follicular helper cells (TFH), CD8 T cells, Gamma delta T cells (Tgd), Cytotoxic cells, T cells, B cells, Tcm, Neutrophils, Regulatory T cells (Treg), and NK CD56dim cells. Only 3 types of immune cells including Th2 cells, NK CD56bright cells, and Th17 cells were negatively correlated with GLT8D2 expression (Fig. 7F and Table S4). Therefore, GLT8D2 expression was significantly correlated with immune infiltration in GC, while the correlation between GLT8D1 and immune cell infiltration is relatively weak.

#### Analyses of the immune checkpoint genes and immune regulators

**Fig. 8:**
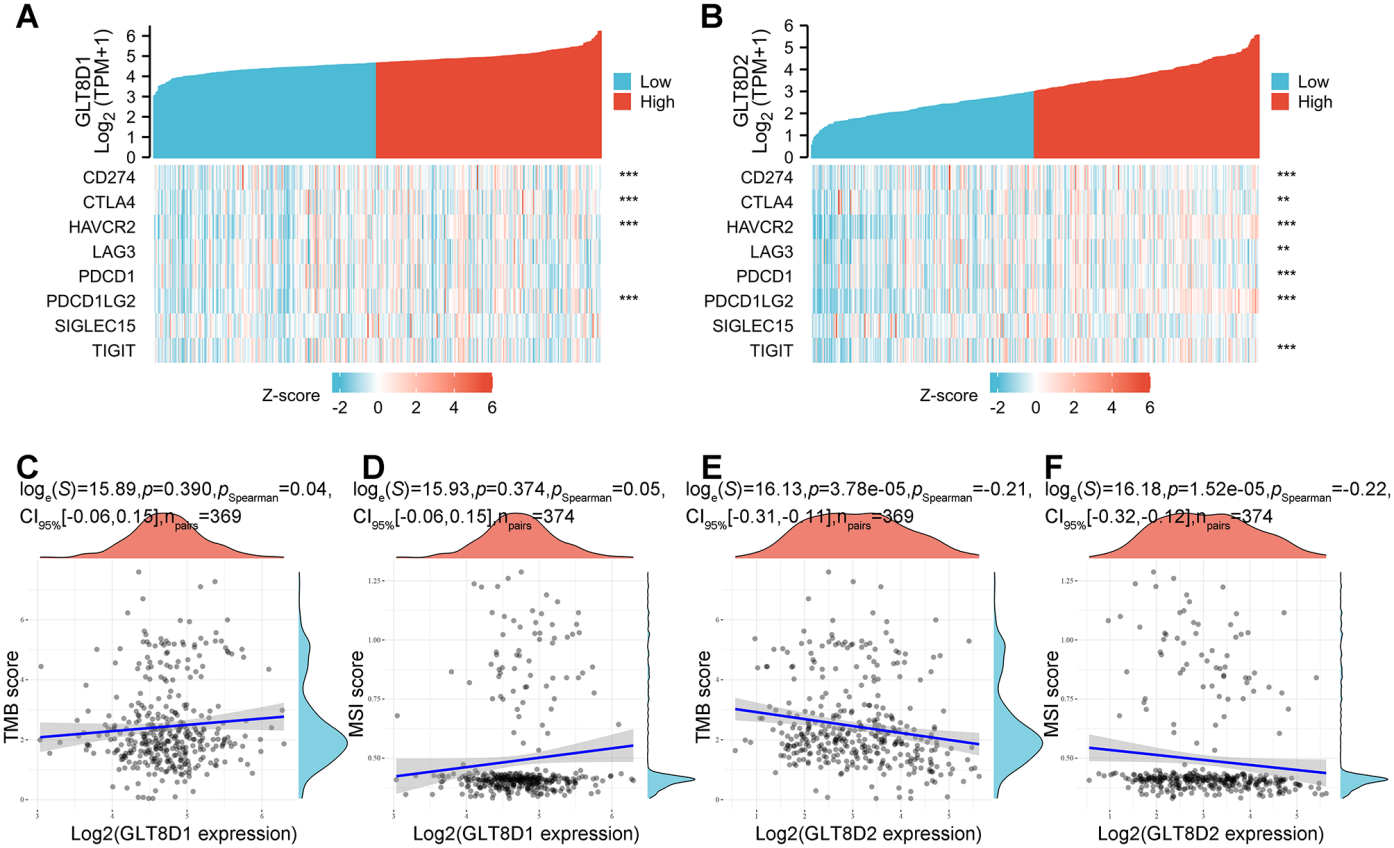
Correlation between GLT8D1/2 expression and immune checkpoint genes and immune regulators TMB/MSI in gastric cancer. **(A, B)** The correlation of GLT8D1 **(A)** and GLT8D2 **(B)** expression with eight immune checkpoint genes. **(C)** Correlation between GLT8D1 and TMB. **(D)** Correlation between GLT8D1 expression and MSI. **(E)** Correlation between GLT8D2 and TMB. **(F)** Correlation between GLT8D1 expression and MSI. TMB, tumor mutational burden; MSI, microsatellite instability. ∗∗ *P* < 0.01, ∗∗∗ *P* < 0.001

Our findings indicated that GLT8D1 expression was significantly and positively correlated with CD274 (PD-L1), CTLA4, HAVCR2, and PDCD1LG2 (PD-L2) (Fig. 8A). GLT8D2 expression was significantly and positively correlated with 7 immune checkpoint genes including CD274 (PD-L1), CTLA4, HAVCR2, LAG3, PDCD1 (PD-1), PDCD1LG2 (PD-L2), and TIGIT, excepting SIGLEC15 (Fig. 8B).

Additionally, we further investigated the relationship between GLT8D1/2 genes and TMB/MSI, two well-known biomarkers for immune response in cancer. No significant correlation was observed between GLT8D1 expression and TMB/MSI (Fig. 8C and D), while GLT8D2 expression was negatively and significantly correlated with both TMB and MSI (Fig. 8E and F). Considering the results above, our study suggested that GLT8D1/2 may affect the prognosis of GC patients through tumor immunity, especially GLT8D2.

### Gene alterations and DNA methylation of GLT8D1/2 in GC

**Fig. 9:**
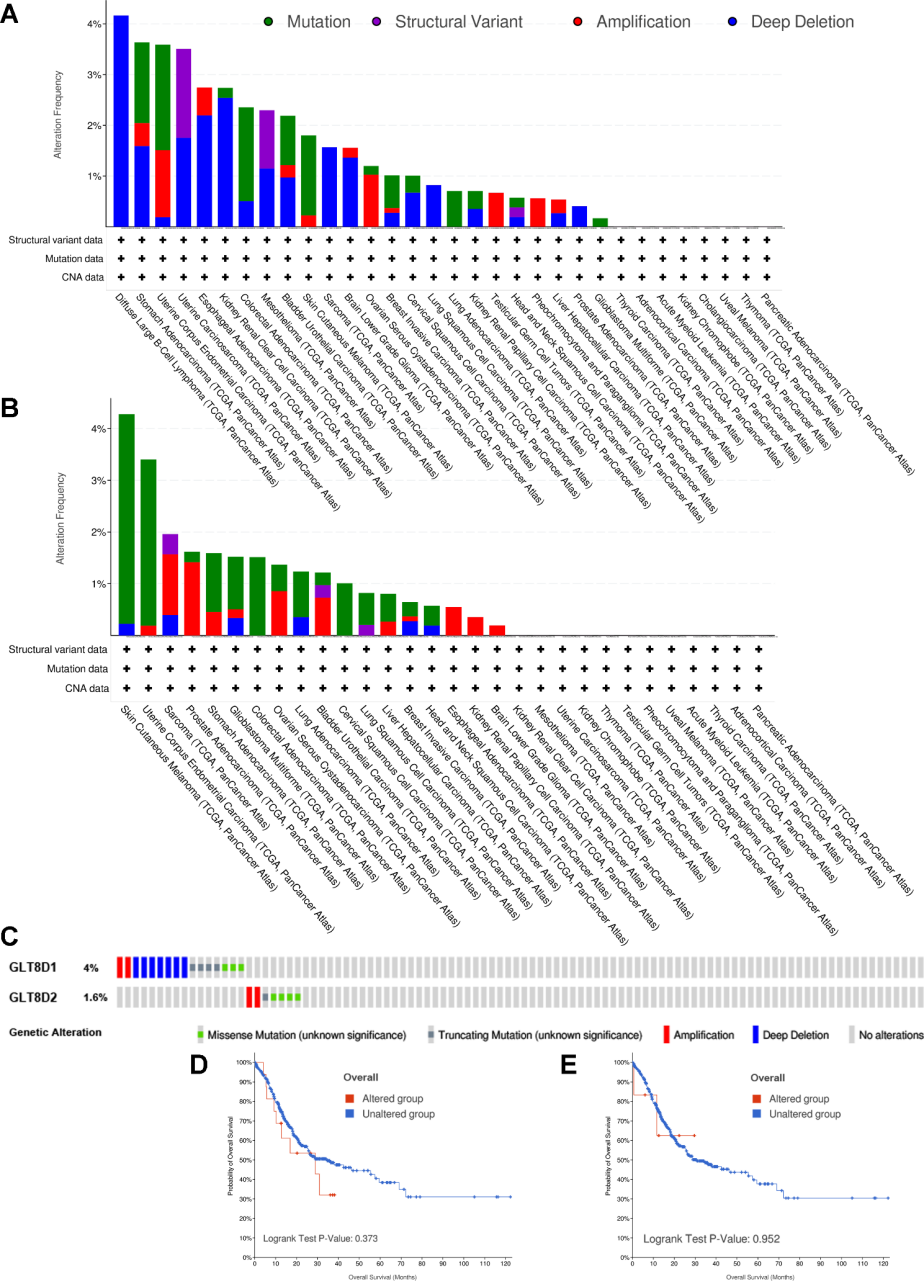
Analysis of GLT8D1/2 genetic alterations. **(A, B)** Total mutations of GLT8D1 **(A)** and GLT8D2 **(B)** in pan-cancer from the cBioPortal database. **(C)** The alteration frequency of GLT8D1/2 with different mutation types in gastric cancer. **(D, E)** The association of genetic alterations in GLT8D1 **(D)** and GLT8D2 **(E)** with OS prognosis of gastric cancer patients. OS, overall survival

We analyzed genetic alterations in GLT8D1/2 and their associations with OS prognosis in GC. As shown in Fig. 9A and B, and 9 C, mutation rates of GLT8D1/2 were 3.64% and 1.59% among 440 GC patients, respectively. Besides, no significant correlations were observed between genetic alterations in GLT8D1/2 and OS of GC patients (Fig. 9D and E).

**Fig. 10:**
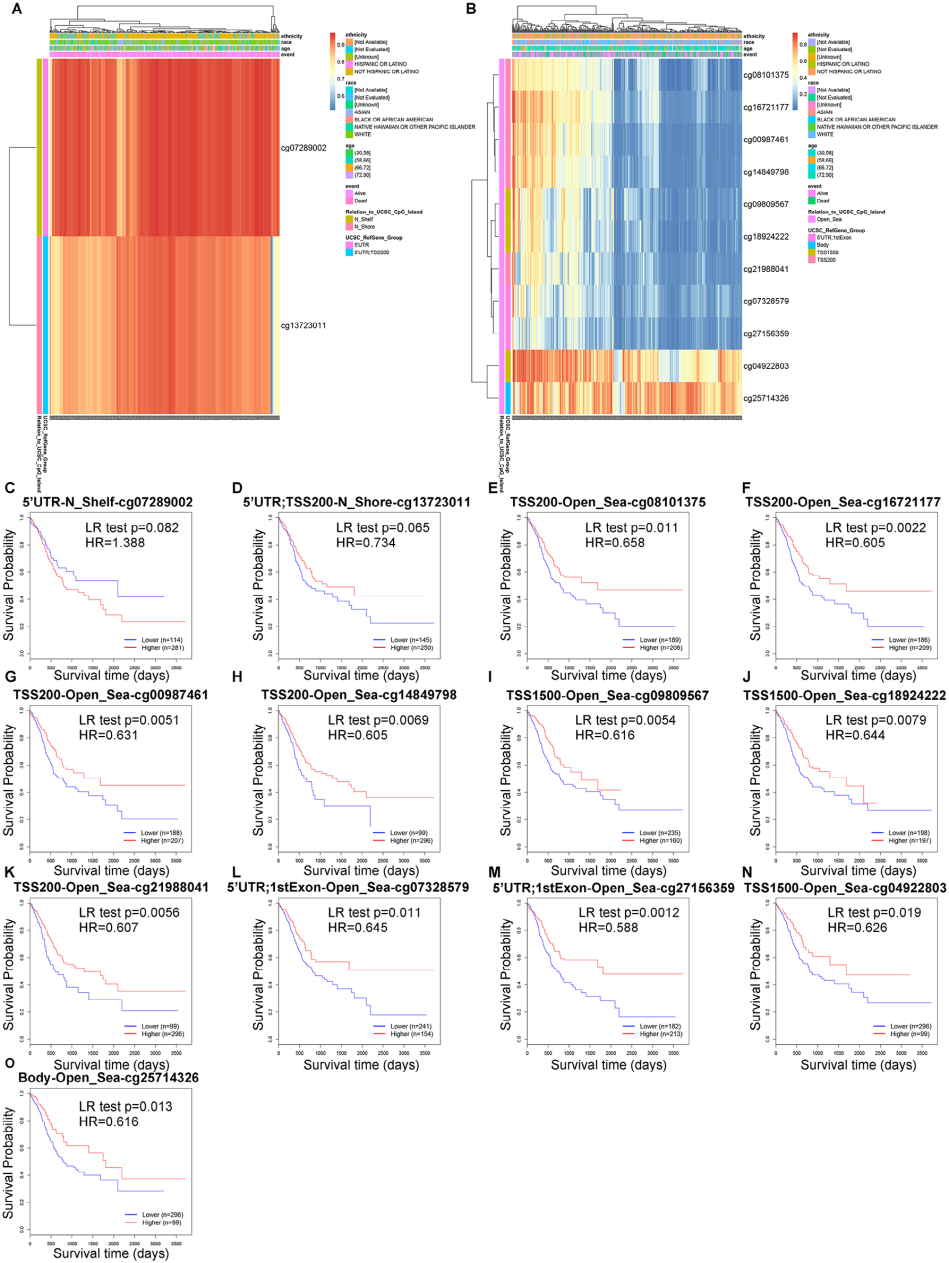
Analysis of GLT8D1/2 methylation levels in gastric cancer based on the MethSurv database. **(A, B)** Heat map depicting clustering of the CpGs methylation levels within GLT8D1 **(A)** and GLT8D2 **(B)** in gastric cancer. Methylation levels (0 = fully unmethylated; 1 = fully methylated) are shown as a continuous variable from a blue to red color. Rows correspond to the CpGs, the columns correspond to the samples. **(C, D)** The Kaplan-Meier survival analysis of the promoter methylation of GLT8D1. **(E-O)** The Kaplan-Meier survival analysis of the promoter methylation of GLT8D2. The red and blue lines of Kaplan-Meier plots indicate higher (β > cut-off) and lower (β < cut-off) methylation patient groups, respectively, dichotomized according to the best cut-off point in MethSurv. HR, hazard ratio; LR, Log-likelihood ratio

The DNA methylation levels of GLT8D1/2 in GC were also analyzed. The results showed that GLT8D1 methylation level is high in GC (Fig. 10A), but there is no significant association between 2 CpG sites of GLT8D1 methylation and OS prognosis of GC patients (Fig. 10 C and 10D). However, GLT8D2 methylation level is generally low in GC (Fig. 10B). We discovered 11 CpG sites located on the CpG island. The survival analyses indicated that GC patients with low GLT8D2 methylation had a poor OS prognosis than that with high methylation levels (*P* < 0.05) (Fig. 10E-O).

## Discussion

GC treatment has significantly developed in recent decades, especially focusing on immunotherapy. However, GC patients’ prognosis remains poor, with a combined 5-year survival rate of about 30% [41, 42]. One of the important reasons is that GC patients at an early stage are not often diagnosed until they have advanced stages due to a lack of specific symptoms and effective early diagnosis. Therefore, there is an urgent need to explore the potential biology mechanism driving GC and reliable biomarkers for improving the diagnostic, prognostic, and therapeutic approaches.

Protein glycosylation, the most abundant post-translational modification, is essential for protein folding, stability, and function and plays critical roles in immune recognition, adhesion, cell signaling, and cell-cell interaction [43, 44]. Aberrant glycosylation is a universal feature of cancer cells correlated with cancer invasion and metastasis. Aberrant glycosylation is probably caused by differential expressions or specific altered activity of glycosyltransferase and glycosidases. These enzymes could act as cancer biomarkers, and cancer-specific changes in glycosyltransferase expression exhibit the most marked and consistent activity alteration in tumorigenesis [45, 46]. Glycosyltransferases are fundamentally involved in multiple biologic processes, such as cell development, migration and invasion, and carcinogenesis [20, 44]. Importantly, aberrant glycosylation pattern due to abnormal glycosyltransferase activity is usually associated with invasion and metastasis of GC [45]. Glycosyltransferase GLT8D1 and GLT8D2 have been reported to be associated with head and neck squamous cell carcinoma, melanomas, glioma, GBM, and ovarian cancer chemoresistance [14–17, 20]. However, until now, the roles and molecular mechanisms of GLT8D1/2 in GC have not been reported.

In the current study, a significant elevation of GLT8D1/2 mRNA expression in GC was found. Results pointed toward a worse overall survival prognosis for GC patients with high GLT8D1/2 expression, which could be independent prognostic factors. The results about GLT8D1 are consistent with previous studies in other tumor types. GLT8D1 overexpression with hypomethylation was reported to act as an oncogene in head and neck squamous cell carcinomas. The gene expression was inversely associated with differential promoter methylation, suggesting that promoter demethylation of GLT8D1 may be a mechanism responsible for gene hyperactivation [14]. GLT8D1 was also confirmed to be upregulated in cutaneous melanomas and might act as a novel prognostic biomarker for an unfavorable prognosis [15]. Moreover, GLT8D1 was confirmed to be significantly upregulated in GBM compared to normal brain tissues and correlated with a worse clinical outcome [17]. Another study showed that GLT8D1 overexpression is associated with more aggressive disease in human gliomas [16]. Only one study implied that overexpression of GLT8D2 confers CDDP resistance to ovarian cancer via activating the FGFR/PI3K/AKT signaling pathway, and suggested that GLT8D2 is a potential therapeutic target for ovarian cancer to enhance platinum response in patients with chemoresistance [20]. Therefore, GLT8D1 and GLT8D2 may be promising therapeutic targets and potential prognostic biomarkers for GC in the future.

Starting with this observation, we further carried out the functional enrichment analyses. GLT8D1 and co-expressed molecules exhibited enrichments of signaling pathways, such as mTOR signaling pathway, cell cycle, protein transferase activity, and nucleocytoplasmic transport based on GO/KEGG enrichment analyses. GSEA exhibited enrichments of Hedgehog, FGF, Notch, and TGFBR pathways. In addition, GLT8D2-related genes showed enrichment of multiple pathways like PI3K-Akt, MAPK, NF-kappa B signaling pathways, cell cycle checkpoints, and pathways in cancer. Notch and mTOR signaling pathways could promote GC cell proliferation [47]. Akt/mTOR signaling pathway is identified to be associated with GC cell apoptosis, decreased phosphorylation levels of Akt and mTOR significantly increase apoptosis [48, 49]. Hedgehog, FGF, Notch, and TGF-β, as key developmental signaling pathways, have been confirmed to play vital roles during regeneration, which can interact with other cellular signaling pathways, such as NF-κB, MAPK, PI3K, and EGF. These developmental pathways may be important therapeutic targets for self-renewal of cancer stem cells and proliferation, and tumor progression [50]. Several pathways like Hedgehog, Notch, NF-κB, and TGF‐β are crucial in EMT, implicated in cancer invasion and metastasis, which made cells acquire stem cell‐like characteristics and resistance to chemotherapy and/or radiotherapy [50, 51]. Therefore, new treatment strategies targeting these pathways are urgently needed to overcome resistance. GLT8D1/2 expression was significantly correlated with multiple signaling pathways or cellular biology involving oncogenesis, cancer development, and clinical prognosis. Importantly, there is a broader crosstalk between these different signaling pathways, and more in-depth research are needed to confirm the exact mechanisms of GLT8D1/2.

It is known that the tumor microenvironment (TME), especially the tumor immune microenvironment (TIME), is a key component of tumor biology, affecting tumor development and prognosis [52]. The TME is a diverse ecosystem containing different types of cells, such as immune cells, fibroblasts, mesenchymal stem cells, and endothelial cells. These cells could affect tumor growth, progression, metastasis, and therapeutic response [53, 54]. CAFs, as the main resource of tumor stroma, are one of the most active and functionally important components of the TME [55, 56]. Accumulating studies confirmed that CAFs could promote tumorigenesis, invasive, metabolism, metastasis, and chemotherapeutic resistance [57]. In the present study, both GLT8D1/2 showed a significant positive correlation with CAFs infiltration in GC, indicating that GLT8D1/2 may affect the prognosis of GC patients through enhancing CAFs infiltration level.

Additionally, GLT8D1 and GLT8D2 were positively correlated with Tcm, Tem, Macrophages, Eosinophils, Neutrophils, and Th1 cells. Tcm and Tem are two subsets of memory T cells (Tm), correlated with the depth of invasion and lymph nodes metastasis of gastric patients. The presence of Tm may improve immune tolerance. Previous study [58] showed that growing numbers of CD8 + Tem may be an indicator of tumor progression. Neutrophils infiltration, alone or in concert with other immune cells, such as Macrophages, Eosinophils, and Mast cells, also lead to tumor development [59], which may provide potential mechanism by which GLT8D1/2 affects prognosis.

Interestingly, GLT8D2 expression was also found to have a broad positive correlation with the infiltration of iDCs, pDCs, DCs, and TReg cells, which indicate that GLT8D2 may play a crucial role in promoting immune cell infiltration within the tumor microenvironment. Although DCs are essential in mounting anti-tumor immune responses, pDCs, as a unique subgroup of DCs, could promote recruitment of Treg to the tumor microenvironment, which leads to immunosuppression, tumor immune escape, and tumor growth [60]. Previous studies reported a positive correlation between circulating pDCs and advanced stages, as well as lymph node metastasis in GC. Furthermore, the accumulation of pDCs predicted poor clinical outcome in GC patients [61, 62]. Th1, Th2, Tregs, and Th17 cells are types of T helper cells involved in tumor regulation by affecting the tumor microenvironment and modulating immune response. Accumulation of Th17 and Tregs in gastric cancer occurred in early disease and then the infiltration of Th17 cells decreased and Treg increased according to the disease progression [63]. Together, GLT8D2 may affect GC development and prognosis by multiple immune cells infiltration, which require further investigation due to complex immunomodulation mechanisms of anti-tumor immune response and immunosuppression.

Moreover, GLT8D1/2 was significantly positively correlated with CD274 (PD-L1), CTLA4, HAVCR2, and PDCD1LG2 (PD-L2). GLT8D2 was also significantly and positively correlated with LAG3, PDCD1 (PD-1), and TIGIT. We observed a significant negative association between GLT8D2 and immunomodulators TMB/MSI. Inhibitors of CTLA-4, PD-L1 and PD-1 receptors are the first drugs of immune checkpoint blockade, which promote T-cell activation. Other immune checkpoint inhibitors are approved or in active preclinical and clinical development [64]. Notably, PD-1/PD-L1 expression, together with TMB and MSI, function as predictive biomarkers for cancer immunotherapy [65]. TMB was defined as a total number of somatic mutations per coding area of a tumor genome. Marabelle et al. [36] indicated that high tissue TMB status identified a subgroup of patients who could have a robust response to anti-PD-1 monoclonal antibody. A substantial portion of patients with high TMB in many disease types might benefit from immunotherapy [64]. High TMB might increase T-cell reactivity due to more neoantigens, which is considered a novel biomarker of sensitivity to immune checkpoint inhibitors and is significantly correlated with clinical benefit of immunotherapy targeting PD-1/PD-L1 and CTLA-4 [36, 66–68]. MSI, as a marker of DNA mismatch repair (dMMR), indicates a condition of genetic hypermutability due to defective dMMR in cancers containing thousands of mutations located in monomorphic microsatellites [65]. MSI is found in sporadic colon, gastric, sporadic endometrial, and most other cancers. Detecting MSI status has prognostic and therapeutic implications in cancers [69]. MSI-high colorectal tumors have improved prognosis compared with microsatellite stable tumors and are more susceptible to immune checkpoint inhibitors, such as PD-1 inhibitors [38, 69, 70]. Patients with MSI-positive tumors are predicted to benefit from novel immunotherapies, so MSI testing across multiple cancer types is needed to expand. Taken together, it may be possible to combine GLT8D1/2 with immune checkpoint genes and TMB/MSI as immunotherapy targets or predictive markers for immunotherapy response in GC. Our findings implied that GLT8D1/2 genes may affect the prognosis of GC patients through tumor immunity, especially GLT8D2.

Genetic alterations play a critical role in tumorigenesis and cancer progression, and clinically relevant tumor gene mutations are increasingly important for genome-directed cancer treatment [71]. GC patients with genetic alterations of oncogenes showed a significantly smaller number of lymph nodes with metastasis and a better prognosis than those without [72]. Our study found that GLT8D1/2 gene alterations occurred in multiple cancer types, among GC samples, GLT8D1 and GLT8D2 were altered in 3.64% and 1.59%, respectively.

In addition to genetic alteration, epigenetic alterations are important in cancer development and progression, which activate growth-promoting pathways and inactivate tumor-suppressive pathways in GC. DNA methylation, one of the most common epigenetic modifications, maybe a vital mechanism of gastric carcinogenesis [72, 73]. Aberrant DNA methylation of a promoter CpG island, as a hallmark of cancer, can induce altered or dysregulated gene expression during tumorigenesis and is associated with cancer progression and prognosis [74, 75]. Our findings indicated a general hypomethylation level of GLT8D2 in GC, and patients with low GLT8D2 methylation had a poor OS prognosis than that with high methylation levels. Therefore, the analysis results indicate that DNA methylation patterns of GLT8D2 may play a role in the development and prognosis of gastric cancer. The methylation levels of GLT8D2 have the potential to serve as a prognostic indicator for overall survival in GC patients. Meanwhile, the methylation patterns of GLT8D1 may not be significantly associated with prognostic indicators.

In this study, bioinformatics analysis was performed using multiple databases. There are still some limitations. Firstly, the present study lacked further experimental validation and exploration of the underlying mechanisms. Secondly, the mechanisms of gene overexpression and oncogenic properties have not been validated. Meanwhile, the causal relationship whether GLT8D1/2 could affect cancer prognosis and response to therapeutic interventions through tumor immunity remains unclear. Therefore, more in-depth studies are needed to validate the roles of GLT8D1/2 in GC.

## Conclusions

To conclude, our study first comprehensively demonstrated that high GLT8D1/2 expression may link to GC pathogenesis and indicate a poor prognosis of GC patients. More importantly, our findings shed light on the potential roles of GLT8D1/2 as prognostic biomarkers and therapeutic targets for immunotherapy in GC.

## Electronic supplementary material

Below is the link to the electronic supplementary material.

Supplementary figure and tables

## Data Availability

The datasets generated and analyzed during the current study are publicly available, including TCGA + GTEx databases (http://xena.ucsc.edu/), UCSC XENA (https://xenabrowser.net/datapages/), Kaplan-Meier Plotter (https://kmplot.com/analysis/index.php?p=service), STRING database (https://cn.string-db.org/), MSigDB Collections (https://www.gsea-msigdb.org/gsea/msigdb/collections.jsp#C2), GEPIA2 (http://gepia2.cancer-pku.cn/), cBioPortal (http://cbioportal.org), TIMER 2.0 (http://timer.cistrome.org/), and MethSurv online tool (https://biit.cs.ut.ee/methsurv/). The datasets used and analyzed during the current study are available from the corresponding author on reasonable request.

## Abbreviations

GC: Gastric cancer
GLT8D1: Glycosyltransferase 8 domain containing 1
GLT8D2: Glycosyltransferase 8 domain containing 2
ALS: Amyotrophic lateral sclerosis
FTD: Frontotemporal dementia
CDDP: Cisplatin
OS: Overall survival
DSS: Disease-specific survival
RFS: Recurrence-free survival
ROC: Receiver operating characteristic curves
AUC: Area under ROC curve
PPI: Protein-protein interaction
CAFs: Cancer-associated fibroblasts
TMB: Tumor mutational burden
MSI: Microsatellite instability
TME: Tumor microenvironment
TIME: Tumor immune microenvironment
dMMR: DNA mismatch repair
GO: Gene Ontology
KEGG: Kyoto Encyclopedia of Genes and Genomes
BP: Biological process
CC: Cellular component
MF: Molecular function
DEGs: Differentially expressed genes
GSEA: Gene set enrichment analysis
Tcm: Central Memory T cell
Tem: Effector Memory T cell
Th1 cells: T helper Type 1 cells
Th2 cells: T helper Type 2 cells
pDC: Plasmacytoid dendritic cells
NK cells: Natural Killer cells
iDC: Immature dendritic cells
DC: Dendritic cells
TFH: T follicular helper cells
Tgd: Gamma delta T cells
TReg: Regulatory T cells
