# Correction: Glycosyltransferase GLT8D1 and GLT8D2 serve as potential prognostic biomarkers correlated with Tumor Immunity in Gastric Cancer

**DOI:** 10.1186/s12920-023-01578-9

**Published:** 2023-06-16

**Authors:** Huimei Xu, Ke Huang, Yimin Lin, Hang Gong, Xueni Ma, Dekui Zhang

**Affiliations:** 1 Department of Gastroenterology, The Second Clinical Medical College of Lanzhou University, Lanzhou, 730030 P.R. China; 2 Lanzhou University Second Hospital, Lanzhou, 730030 P.R. China; 3 School of Basic Medical Sciences, Lanzhou University, Lanzhou, 730030 P.R. China; 4 Key Laboratory of Digestive Diseases of Lanzhou University Second Hospital, Lanzhou, 730030 P.R. China

**Correction: BMC Med Genomics 16, 123 (2023)**

10.1186/s12920-023-01559-y

Following publication of the original article [1], the authors reported errors in the numbering of the in-text citations from number 16 onwards. The original article [1] has been corrected.

## References

[1] Xu H, Huang K, Lin Y (2023). Glycosyltransferase GLT8D1 and GLT8D2 serve as potential prognostic biomarkers correlated with Tumor Immunity in Gastric Cancer. BMC Med Genomics.

