# Supplementary material for: Glycosyltransferase GLT8D1 and GLT8D2 serve as potential prognostic biomarkers correlated with Tumor Immunity in Gastric Cancer
Source: BMC Med Genomics. 2023 Jun 5;16:123. doi: 10.1186/s12920-023-01559-y (PMC10242987; doi:10.1186/s12920-023-01559-y)
Supplement: Supplementary file 1 — Supplementary figure and tables [file 12920_2023_1559_MOESM1_ESM.docx]

Supplementary Material


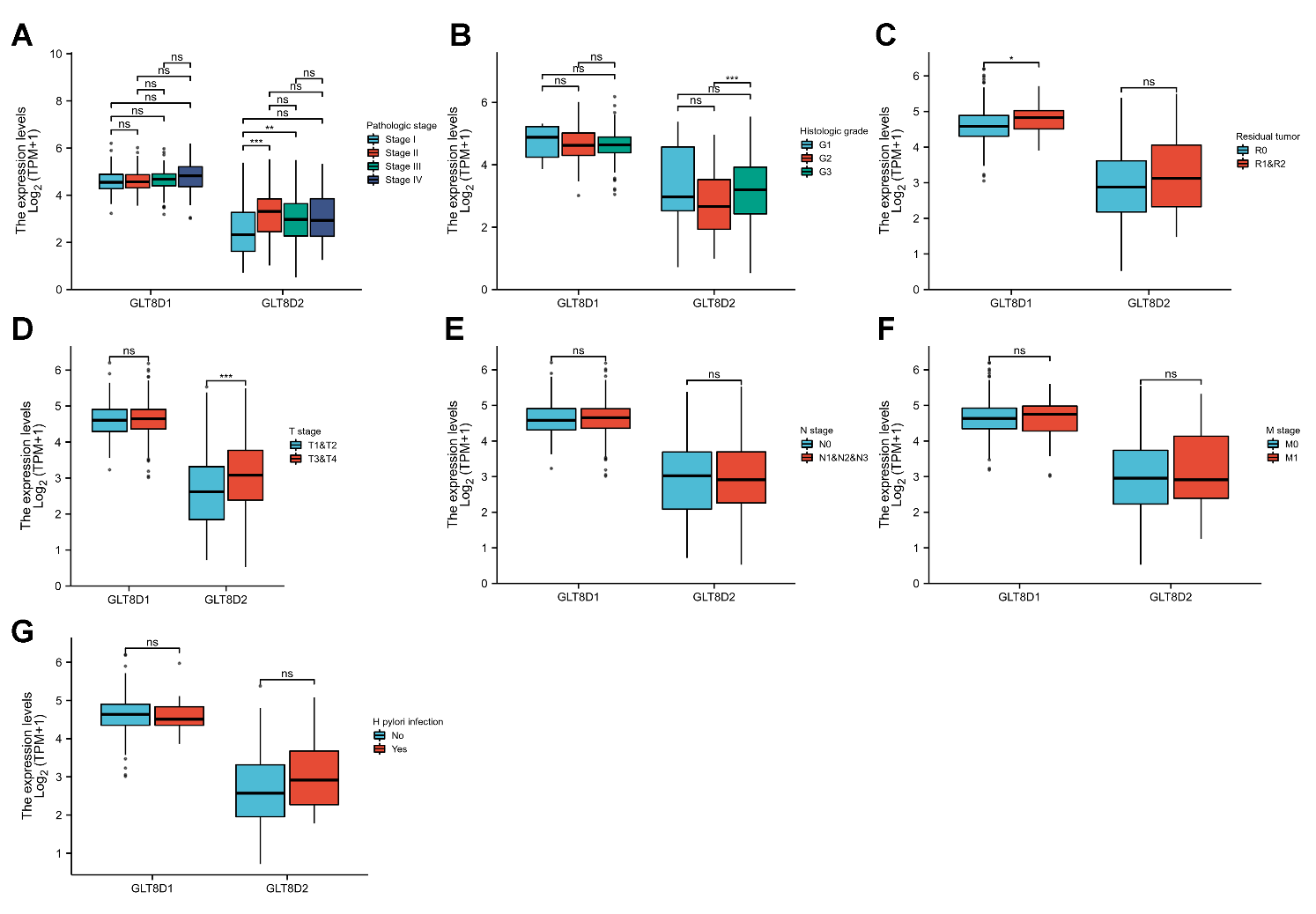


**Figure S1.** Association between GLT8D1/2 expression and clinical characteristics, including pathologic stage (**A**), histologic grade (**B**), residual tumor (**C**), T stage (**D**), N stage (**E**), M stage (**F**), and Helicobacter pylori infection (**G**) in gastric cancer. ns, no significance. **P*<0.05; ** *P*<0.01; *** *P*<0.001.

| **Table S1.** GO/KEGG enrichment analyses of GLT8D1-related genes. | | | | | | | |
| --- | --- | --- | --- | --- | --- | --- | --- |
| Ontology | ID | Description | Gene Ratio | Bg Ratio | *P* value | *p*.adjust | Q value |
| BP | GO:0016569 | covalent chromatin modification | 212/4049 | 474/18670 | 8.03e-30 | 2.48e-26 | 2.08e-26 |
| BP | GO:0016570 | histone modification | 205/4049 | 454/18670 | 1.51e-29 | 3.10e-26 | 2.60e-26 |
| BP | GO:0006913 | nucleocytoplasmic transport | 159/4049 | 343/18670 | 1.20e-24 | 1.85e-21 | 1.56e-21 |
| CC | GO:0000151 | ubiquitin ligase complex | 139/4208 | 282/19717 | 1.37e-25 | 5.11e-23 | 3.31e-23 |
| CC | GO:0098687 | chromosomal region | 146/4208 | 349/19717 | 2.69e-18 | 6.69e-16 | 4.33e-16 |
| CC | GO:0031248 | protein acetyltransferase complex | 57/4208 | 95/19717 | 3.08e-16 | 3.84e-14 | 2.48e-14 |
| MF | GO:0019787 | ubiquitin-like protein transferase activity | 193/4084 | 407/17697 | 1.17e-27 | 1.28e-24 | 1.02e-24 |
| MF | GO:0004842 | ubiquitin-protein transferase activity | 182/4084 | 382/17697 | 1.90e-26 | 1.04e-23 | 8.30e-24 |
| MF | GO:0140098 | catalytic activity, acting on RNA | 179/4084 | 386/17697 | 2.51e-24 | 9.15e-22 | 7.30e-22 |
| KEGG | hsa04120 | Ubiquitin mediated proteolysis | 79/1742 | 140/8076 | 1.34e-19 | 4.24e-17 | 3.57e-17 |
| KEGG | hsa04150 | mTOR signaling pathway | 57/1742 | 155/8076 | 9.12e-06 | 2.40e-04 | 2.02e-04 |
| KEGG | hsa04110 | Cell cycle | 44/1742 | 124/8076 | 2.39e-04 | 0.005 | 0.004 |
| GO, Gene Ontology; KEGG, Kyoto encyclopedia of genes and genomes; BP, biological process; CC, cellular component; MF, molecular function. | | | | | | | |

| **Table S2.** GO/KEGG enrichment analyses of GLT8D2-related genes. | | | | | | | |
| --- | --- | --- | --- | --- | --- | --- | --- |
| Ontology | ID | Description | Gene Ratio | Bg Ratio | *P* value | *p*.adjust | Q value |
| BP | GO:0030198 | extracellular matrix organization | 188/3130 | 368/18670 | 8.51e-53 | 5.23e-49 | 3.41e-49 |
| BP | GO:0043062 | extracellular structure organization | 201/3130 | 422/18670 | 3.64e-50 | 1.12e-46 | 7.30e-47 |
| BP | GO:0031589 | cell-substrate adhesion | 153/3130 | 354/18670 | 3.00e-32 | 6.15e-29 | 4.01e-29 |
| CC | GO:0062023 | collagen-containing extracellular matrix | 211/3297 | 406/19717 | 5.21e-61 | 3.56e-58 | 2.61e-58 |
| CC | GO:0005924 | cell-substrate adherens junction | 149/3297 | 408/19717 | 1.80e-22 | 3.97e-20 | 2.91e-20 |
| CC | GO:0030055 | cell-substrate junction | 150/3297 | 412/19717 | 1.83e-22 | 3.97e-20 | 2.91e-20 |
| MF | GO:0005201 | extracellular matrix structural constituent | 106/3108 | 163/17697 | 2.09e-41 | 2.28e-38 | 1.85e-38 |
| MF | GO:0005539 | glycosaminoglycan binding | 110/3108 | 229/17697 | 1.58e-26 | 8.58e-24 | 6.99e-24 |
| MF | GO:0005178 | integrin binding | 72/3108 | 132/17697 | 6.31e-22 | 2.29e-19 | 1.86e-19 |
| KEGG | hsa04151 | PI3K-Akt signaling pathway | 117/1440 | 354/8076 | 1.16e-12 | 9.10e-11 | 5.95e-11 |
| KEGG | hsa04010 | MAPK signaling pathway | 83/1440 | 294/8076 | 5.05e-06 | 4.80e-05 | 3.14e-05 |
| KEGG | hsa04064 | NF-kappa B signaling pathway | 31/1440 | 104/8076 | 0.002 | 0.008 | 0.005 |
| GO, Gene Ontology; KEGG, Kyoto encyclopedia of genes and genomes; BP, biological process; CC, cellular component; MF, molecular function. | | | | | | | |

| **Table S3** Correlation between GLT8D1 expression and immune cell infiltration. | | |
| --- | --- | --- |
| Immune cells type | Correlation coefficient (Spearman) | *P* value (Spearman) |
| T helper cells | 0.217 | <0.001 |
| Tcm | 0.214 | <0.001 |
| Tem | 0.164 | 0.001 |
| Macrophages | 0.159 | 0.002 |
| Eosinophils | 0.153 | 0.003 |
| Neutrophils | 0.116 | 0.025 |
| Th2 cells | 0.113 | 0.028 |
| Th1 cells | 0.111 | 0.031 |
| iDC | 0.090 | 0.083 |
| NK CD56dim cells | -0.086 | 0.095 |
| B cells | -0.085 | 0.099 |
| Cytotoxic cells | -0.084 | 0.106 |
| T cells | -0.054 | 0.298 |
| Tgd | -0.034 | 0.512 |
| Th17 cells | 0.014 | 0.792 |
| CD8 T cells | -0.013 | 0.803 |
| aDC | -0.012 | 0.820 |
| TReg | -0.011 | 0.832 |
| Mast cells | -0.011 | 0.835 |
| DC | 0.011 | 0.830 |
| NK CD56bright cells | -0.009 | 0.869 |
| NK cells | -0.007 | 0.894 |
| TFH | -0.005 | 0.917 |
| pDC | -0.149 | 0.004 |

| **Table S4** Correlation between GLT8D2 expression and immune cell infiltration. | | |
| --- | --- | --- |
| Immune cells type | Correlation coefficient (Spearman) | *P* value (Spearman) |
| Macrophages | 0.619 | <0.001 |
| NK cells | 0.619 | <0.001 |
| Mast cells | 0.522 | <0.001 |
| iDC | 0.501 | <0.001 |
| Tem | 0.476 | <0.001 |
| pDC | 0.457 | <0.001 |
| DC | 0.423 | <0.001 |
| Eosinophils | 0.395 | <0.001 |
| Th1 cells | 0.389 | <0.001 |
| TFH | 0.328 | <0.001 |
| CD8 T cells | 0.324 | <0.001 |
| Tgd | 0.324 | <0.001 |
| Cytotoxic cells | 0.290 | <0.001 |
| T cells | 0.247 | <0.001 |
| B cells | 0.235 | <0.001 |
| Tcm | 0.215 | <0.001 |
| Neutrophils | 0.204 | <0.001 |
| TReg | 0.183 | <0.001 |
| NK CD56dim cells | 0.113 | 0.029 |
| aDC | 0.087 | 0.091 |
| T helper cells | 0.011 | 0.825 |
| Th2 cells | -0.106 | 0.040 |
| NK CD56bright cells | -0.155 | 0.003 |
| Th17 cells | -0.215 | <0.001 |
